# Promoter methylation status of the tumor suppressor gene SOX11 is associated with cell growth and invasion in nasopharyngeal carcinoma

**DOI:** 10.1186/1475-2867-13-109

**Published:** 2013-11-05

**Authors:** Song Zhang, Shuo Li, Jin-Liang Gao

**Affiliations:** 1 Department of Otolaryngology, Guang Ming New District People’s Hospital of Shenzhen, Song Bai Road 339#, Shenzhen, Guang Dong TX 518106, PR China; 2 Department of Otolaryngology, Nanshan People’s Hospital of Shenzhen, Tao Yuan Road 89#, Shenzhen, Guang Dong TX 518052, PR China

**Keywords:** SOX11 gene, Methylation, Nasopharyngeal carcinoma, CNE2 cell line

## Abstract

**Background:**

The transcription factor SOX11 is one of members of the SRY box-containing (SOX) family emerging as important transcriptional regulators. In recent years, up-regulation of SOX11 has been detected in various types of solid tumors. In this study, the effects of promoter methylation of the SOX11 gene on SOX11 expression and cell growth and invasion of nasopharyngeal carcinoma were investigated.

**Methods:**

In this study,methylation-specific PCR and real time quantitative PCR have been applied to investigate the effect of promoter methylation of the SOX11 gene on SOX11 expression in the nasopharyngeal carcinoma and chronic inflammation tissues. The nasopharyngeal carcinoma cell line (CNE2) was treated with 5-aza-2'-deoxycytidine. The effect of promoter methylation of SOX11 on growth and invasion of nasopharyngeal carcinoma cells was detected with MTT test and Boyden chamber Matrigel invasion assay.

**Results:**

No or weak expression of SOX11 mRNA was detected in the nasopharyngeal carcinoma tissues of SOX11 gene promoter methylation. Strong expression of SOX11 mRNA was detected in the nasopharyngeal carcinoma tissues of SOX11 gene promoter unmethylation and chronic inflammation tissues of pharynx nasalis. SOX11 mRNA and protein were re-expressed, SOX11 gene was demethylated, and growth and invasion of cells were inhibited in CNE2 cell line after 5-aza-2'-deoxycytidine treatment.

**Conclusions:**

The results of the study indicate that expression of SOX11 mRNA and protein were related to SOX11 gene methylation status. SOX11 gene methylation may be plays a role in growth and invasion of nasopharyngeal carcinoma cells.

## Introduction

Nasopharyngeal carcinoma (NPC) is a common tumor in the head and neck. There are a high incidence of NPC in south of China. Its pathogenesis is not very clear. It may be related to a variety of factors. In recent years, epigenesis of gene attracted much attention from researchers. Abnormality of DNA methylation is an important mechanism of epigenetic regulation. Methylation status of gene promoter is related to gene activity [1,2]. DNA methylation plays an important role in tumorigenesis. CpG island methylation of tumor suppressor gene resulted in inactivation of the gene transcription has become an important part of cancer epigenetics research. Multiple tumor suppressor genes inactivated by promoter CpG island methylation have been found in a variety of tumor tissues and cells. Such as promoter hypermethylation and BRCA1 inactivation in sporadic breast and ovarian tumors [3], hypermethylation of the APC (adenomatous polyposis coli) gene promoter region is involved in human colorectal carcinoma [4], incidence and functional consequences of hMLH1 promoter hypemethylation in colorectal carcinoma[5], hypermethylation around the promoter may be a mechanism of E-cadherin inactivation in human carcinomas [6]. The transcription factor SOX11 is one of members of the SRY box-containing (SOX) family emerging as important transcriptional regulators, which as a whole controls cell fate and differentiation [7]. Twenty SOX genes have been identified in mouse and human genomes. All SOX genes contain a DNA-binding high mobility group (HMG) domain and protein specific domains implicated in activation and repression of gene transcription [8]. It has been found that SOX11 plays an important role in the development of nervous system and adult neurogenesis [9,10]. SOX11 up-regulation has been detected in various types of solid tumors, such as gliomas and epithelial ovarian tumors [11,12]. Vegliante found that SOX11 expression is related to methylation of SOX11 gene promoter in lymphoid neoplasms [13].

In the present study, we have performed on methylation of SOX11 gene, inclouding DNA methylation in the tissues of nasopharyngeal carcinoma and DNA demethylation in the CNE2 cell line (human nasopharyngeal carcinoma cell line). The findings shows that weak expression of SOX11 is related to methylation of SOX11 gene promoter in the tissues of nasopharyngeal carcinoma, and SOX11 re-expression is associated with demethylation of SOX11 gene by 5-aza-2'-deoxycytidine treated in CNE2 cells.

## Material and methods

### Clinical material

Fifty-six tissues specimens of pharynx nasalis were included in the study. All the biopsies were obtained from patients with consent before treatment at the Department of Otolaryngology of Guangming New District People’s Hospital of Shenzhen. The ratio of male patients and female patients is 4.6 to 1. The age range was 16–62 years with a mean age of 49 years. All specimens were subjected to histological diagnosis by a pathologist. There are forty-three nasopharyngeal carcinoma (NPC) and thirteen chronic inflammation tissues. 43 nasopharyngeal carcinoma tissues are all undifferentiated nasopharyngeal carcinoma. On the basis of TNM stage classification (UICC 2002), 7 (16.3%) patients had stage I disease, 13 (30.2%) patients had stage II disease, 11 (25.6%) patients had stage III disease, 12(27.9%) patients had stage IV disease. As for lymph node metastasis in the neck, 29 patients had lymph node metastasis, and 14 patients had no lymph node metastasis. No chemotherapy or radiotherapy was given to patients with nasopharyngeal carcinoma before biopsy.

### Analysis of methylation status of SOX11 gene promoter

The methylation status of the SOX11 gene promoter was determined by chemical modification with the Methylation-Gold Kit (Zymo Research, Los Angeles, USA) according to the manufacturer’s protocol and the methylation-specific PCR (MSP) procedure. Primer sequences for the methylated (M) sequence: 5'- CGTGTTCGAGTGTGTGTATTC-3'(sense),5'-GAAACGAACCGAAATAATTCG -3' (antisense);For the unmethylated (U)sequence:5'-GTGTGTTTGAGTGTGTGTATTTGG-3' (sense), 5'-CAAAACAAACCAAAATAATTCA-3' (antisense). Amplification was carried out in a Life Express Thermal Cycler for 35 cycles. The annealing temperature for both the unmethylated and methylated reactions was 56°C. The PCR products were analyzed on a 2% agarose gel.

### RNA isolation and real-time quantitative PCR

The total RNA was isolated from tissues or cells by using Trizol regents. Reverse transcription was performed with Revert Aid First Strand cDNA Synthesis Kit. The cDNA was amplified by following TOYOBO THUNDERBIRD SYBR qPCR Mix kit with the following primer specific either for SOX11 or the house-keeping gene β-actin (primers were synthesized by Invitrogen Biotechnology Co., LTD). β-actin: 5'-GTCCACCGCAAATGCTTCTA-3'and5'- TGCTGTCACCTTCACCGTTC-3',SOX11:5'-AAGAACATCACCAAGCAGCACC-3' and 5'-TGTGA ACACCAGGTCGGAGAAG-3'. Real-time PCR products were detected using SLAN Fluorescence Quantitative PCR Detection System. The β-actin gene was used as internal control.

### Western blot analysis

Total nuclear extracts were isolated and analyzed on a SDS-polyacrylamide gel and transferred onto a polyvinylidene difluoride membrane. Immunoblotting was performed using a sheep polyclonal antibody SOX11 and an anti-β-actin antibody. The membranes were washed with Tris-buffered saline and then incubated with a 1:3000 dilution of secondary antibodies. The proteins were visualized by using a chemiluminescence detection kit from Perkin-Elmer.

### Cell culture

The CNE2 cell line, a NPC cell line, was obtained from the China Center for Type Culture Collection. CNE2 cell line was cultured in RPMI-1640 medium (HyClone, Sout Logan, UT) supplemented with 10% (v/v) fetal bovine serum (Sijiqing Biological Engineering Materials Co, Hangzhou, China) at 37°C in 5% CO2.

### MTT test

The proliferation assays were performed by MTT test. The CNE2 cell lines were digested using 0.25% trypsin when the cells were in the logarithmic phase of growth. Then, the cell lines were seeded at a concentration of 1 × 10^5^ cells/ml. The cells were seeded onto 96-well plates at a density of 1 × 10^4^ cells/well in triplicates, and treatment for 24 hours to allow the cells to attach. Then, the medium containing 5-aza-2'-deoxycytidine (5-aza-cdr, 0、0.5、1、5、10、20、40、80、160 μmol/L) was added in each well in 96-well plates. Meanwhile, zeroing wells were arranged in these plates. 50 μl of MTT solution (5 mg/mL) was added in each well after the cells have been cultured for 24 hours. The clear supernatant liquid was blotted and 200 μl of dimethyl sulphoxide (DMSO) was added into each well 2 hours later. The light absorption of solution at 570 nm was determined by using a microplate reader. The inhibition ratio of the drug to the cells was calculated with a formula(IR = (1- experimental group)/control group × 100%.).

### Boyden chamber Matrigel invasion assay

The invasive capacity of control group (without 5-aza-cdr) and experimental group (with 80 μmol/l 5-aza-cdr) of CNE2 cells had been examined by using two compartments: Boyden chambers assay (Corning incorporated, New York, USA) and Matrigel basement membrane matrix (BD Biosciences, New Jersey, USA). All cells were analyzed for their viability, and an equal number of viable cells (10^5^) was added to the upper chamber and allowed to invade through the Matrigel onto the filters for 24 hours. At the end of the incubation period, the filters were washed, fixed, and stained. The invading cells were then examined and counted in 10 randomly selected fields under a light microscope at × 400 Magnification. Then,ten random fields for each set of experiments were analyzed and the average number of cells invaded was calculated.

### Statistical analysis

All statistical analysis was performed using the Statistical Package for Social Sciences (SPSS,version17.0). Chi-square test was used to assess the difference of SOX11 gene methylation between with lymph node metastasis and without lymph node metastasis in the neck and among each TNM stage in the nasopharyngeal carcinoma. The *t* test was used to assess the difference of invasion capacity of CNE2 cells before and after 5-aza-cdr treatment. A *P* value less than 0.05 was considered statistically significant.

## Results

### Methylation status of SOX11 gene in nasopharyngeal carcinoma and chronic inflammation tissues

**Figure 1:**
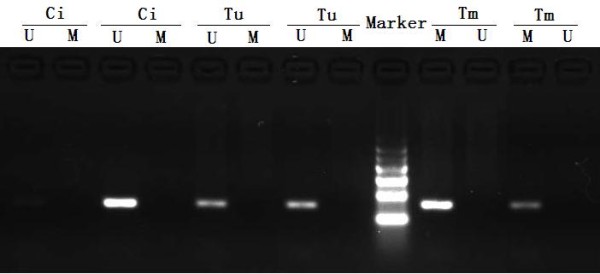
**Methylation status of SOX11 gene in the nasopharyngeal carcinoma and chronic inflammation tissues.** Lane M, Amplified product with primers recognizing methylated sequences; Lane U, Amplified product with primers recognizing unmethylated sequences. Tm: SOX11 gene methylated nasopharyngeal carcinoma tissues; Tu: SOX11 gene unmethylated nasopharyngeal carcinoma tissues; Ci: Chronic inflammation tissues in pharynx nasalis. The results showed that only methylated product was amplified in the nasopharyngeal carcinoma tissues with SOX11 gene promoter methylation, only unmethylated product was amplified in the chronic inflammation tissues and nasopharyngeal carcinoma tissues with SOX11 gene promoter unmethylation.

SOX11 gene promoter methylation was found in 29 of 43 (67.4%) nasopharyngeal carcinoma tissues. None of 13 chronic inflammation tissues of pharynx nasalis showed SOX11 gene promoter methylation (Figure 1). Chi-square test showed that there was no significant difference in methylation rate of the SOX11 gene promoter among the samples from patients with nasopharyngeal carcinoma in different TNM stages. However, the methylation rate of the SOX11 gene promoter in the nasopharyngeal carcinoma tissues from patients with lymph node metastasis is significantly higher than nasopharyngeal carcinoma tissues from patients without lymph node metastasis (Table 1).

**Table 1:** Clinical characteristics in the total cohort and the SOX11 gene methylation positive subgroup

**History**	**N total**	**N SOX11 gene methylation positive**
Chronic inflammation tissues	13	0
Nasopharyngeal carcinoma stage *	43	29
I	7	5
II	13	8
III	11	8
IV	12	8
Lymph node metastasis **		
Positive	29	25
Negative	14	4

* X^2^ = 1.930, *P* = 0.587.

** X^2^ = 5.233, *P* = 0.000.

### Expression of SOX11 mRNA in nasopharyngeal carcinoma and chronic inflammation tissues

**Figure 2:**
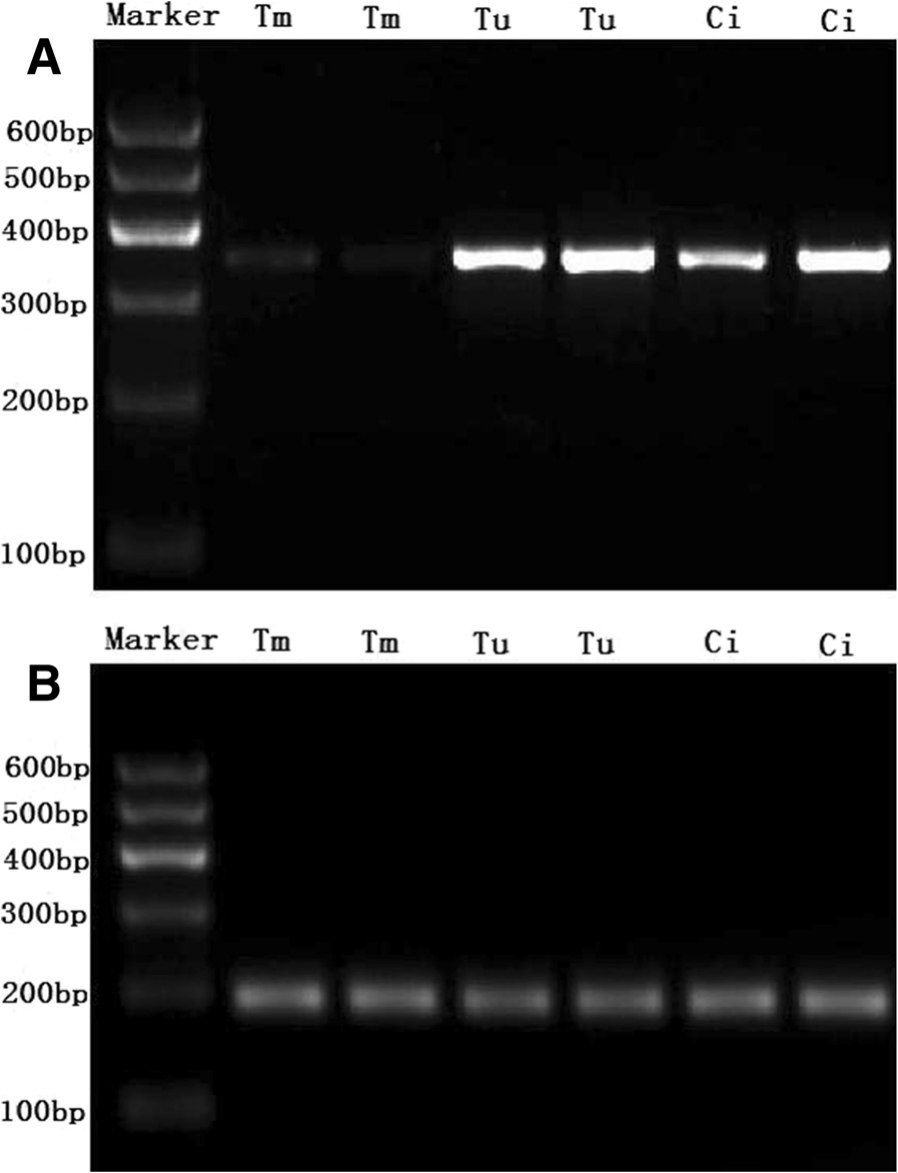
**SOX11 mRNA expression in the nasopharyngeal carcinoma and chronic inflammation tissues. A,** Electrophoretogram of SOX11 mRNA expression; **B,** Electrophoretogram of β-actin mRNA expression; Tm: SOX11 gene methylated nasopharyngeal carcinoma tissues; Tu: SOX11 gene unmethylated nasopharyngeal carcinoma tissues; Ci: Chronic inflammation tissues in pharynx nasalis. It showed that weak expression of SOX11 mRNA was found in the nasopharyngeal carcinoma tissues with SOX11 gene promoter methylation, strong expression of SOX11 mRNA was found in the chronic inflammation tissues and nasopharyngeal carcinoma tissues with SOX11 gene promoter unmethylation.

No expression or very weak expression of SOX11 mRNA was detected in the nasopharyngeal carcinoma tissues with SOX11 gene promoter methylation. Strong expression of SOX11 mRNA was found in the nasopharyngeal carcinoma tissues with SOX11 gene promoter unmethylation and chronic inflammation tissues of pharynx nasalis (Figure 2).

### Expression of SOX11 protein in nasopharyngeal carcinoma and chronic inflammation tissues

**Figure 3:**
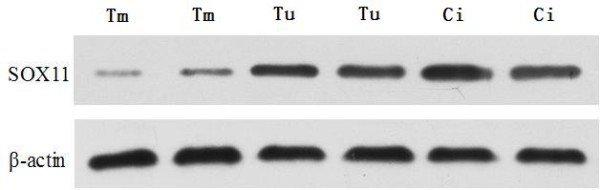
**SOX11 protein expression in the nasopharyngeal carcinoma and chronic inflammation tissues.** Tm: SOX11 gene methylated nasopharyngeal carcinoma tissues; Tu: SOX11 gene unmethylated nasopharyngeal carcinoma tissues; Ci: Chronic inflammation tissues in pharynx nasalis. The electrophoretogram showed that weak expression of SOX11 protein was found in the nasopharyngeal carcinoma tissues with SOX11 gene promoter methylation, strong expression of SOX11 protein was found in the chronic inflammation tissues and nasopharyngeal carcinoma tissues with SOX11 gene promoter unmethylation.

Weak expression of SOX11 protein was detected in the nasopharyngeal carcinoma tissues with SOX11 gene promoter methylation. Strong expression of SOX11 protein was showed in the nasopharyngeal carcinoma tissues with SOX11 gene promoter unmethylation and chronic inflammation tissues of pharynx nasalis (Figure 3).

### MTT test

**Figure 4:**
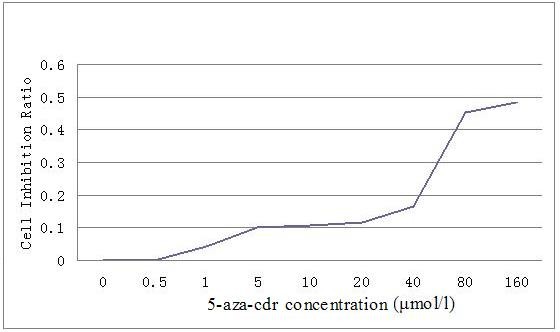
**Growth inhibition plot of CNE2 cells after treated by 5-aza-cdr.** The plot showed that CNE2 cell growth inhibition is more and more obvious with the increase of 5-aza-cdr concentration. When the 5-aza-cdr concentration is 80 μmol/L, the inhibition rate of CNE2 cells growth ratio is almost 50%.

The MTT test showed that the inhibition of cells growth is more and more obvious with the increase of drug concentration. When the drug concentration is 80 μmol/L, the inhibition rate of cells growth is almost 50% (Figure 4).

### Effect of 5-aza-cdr on invasion capacity of CNE2 cells

**Figure 5:**
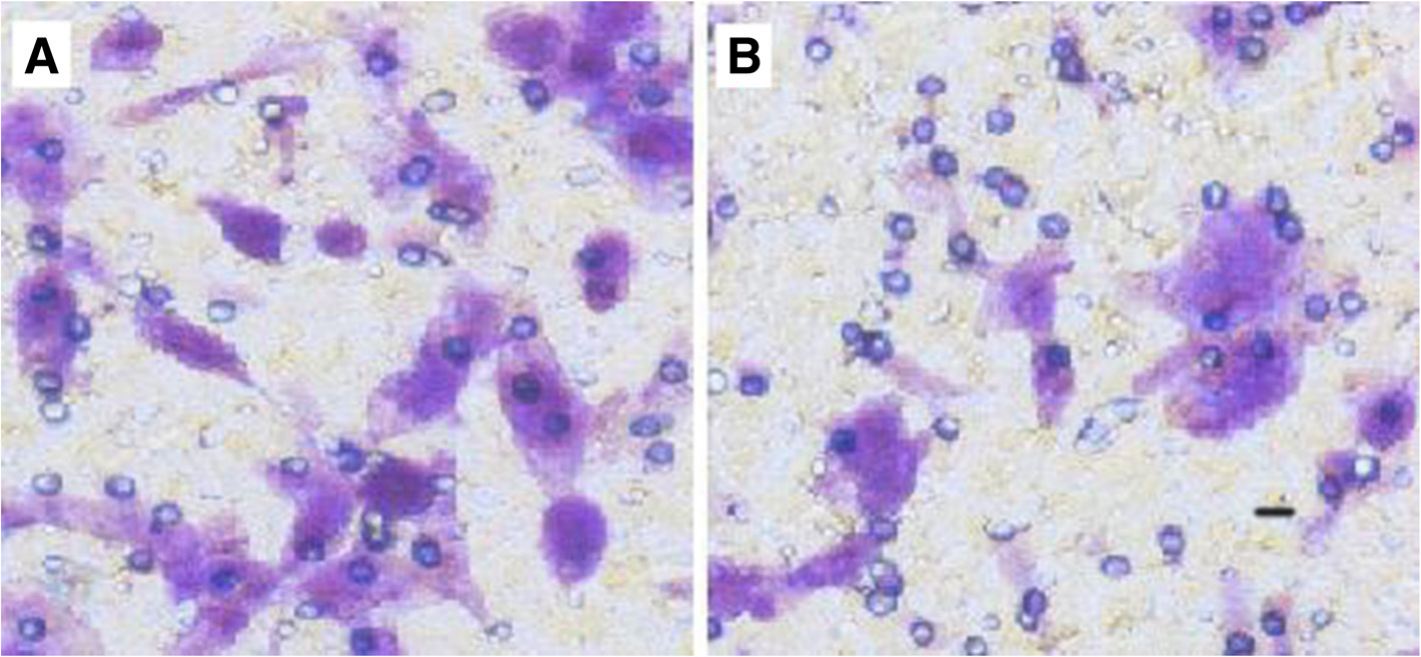
**Effect of 5-aza-cdr on CNE2 cells invasion capacity. A,** CNE2 cells were not treated by any drugs; **B,** CNE2 cells were treated by 80 μmol/L 5-aza-cdr. Invasive capacity of CNE2 cells treated with 5-aza-cdr was significantly decreased than CNE2 cells without any treatment.

The invasive capacity of control group (without 5-aza-cdr) and experimental group (treatment with 5-aza-cdr) CNE2 cells had been examined by using Boyden chamber Matrigel invasion assay. As expected, the invading cells number was significantly decreased in the CNE2 cells treated with 5-aza-cdr(=8.40 ± 1.26) than the CNE2 cells without any treatment(=12.10 ± 1.20, *t* = 6.718, *p* = 0.000). These studies show that invasive capacity of CNE2 cells treated with 5-aza-cdr was significantly decreased than CNE2 cells without any treatment (Figure 5).

### Effect of 5-aza-cdr on methylation of SOX11 gene in the CNE2 cells

**Figure 6:**
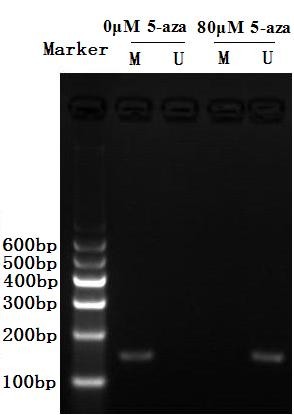
**Effect of 5-aza-cdr on SOX11 gene methylation in the CNE2 cells.** Lane M, Amplified product with primers recognizing methylated sequences; Lane U, Amplified product with primers recognizing unmethylated sequences. Only methylated product was amplified in the CNE2 cells without any treatment. Only unmethylated product was amplified in the CNE2 cells after treated by 5-aza-cdr. It showed that SOX11 gene was demethylated by 5-aza-cdr in the CNE2 cells.

In order to detect the effect of 5-aza-cdr on methylation of SOX11 gene in the CNE2 cells, 10^5^ CNE2 cells were seeded onto 6-well plates in each well and incubated for 24 hours. Then, the medium containing 5-aza-cdr (80 μM) was added randomly to 3 wells. The other 3 wells were not added anything. All CNE2 cells in 6-well plates were digested using 0.25% trypsin after incubating 48 hours. The methylation status of SOX11 gene was detected by using methylation-specific PCR in the CNE2 cells. The results showed that SOX11 gene was demethylated after treating with 5-aza-cdr in the CNE2 cells (Figure 6).

### Effect of 5-aza-cdr on expression of SOX11 mRNA and protein in the CNE2 cells

**Figure 7:**
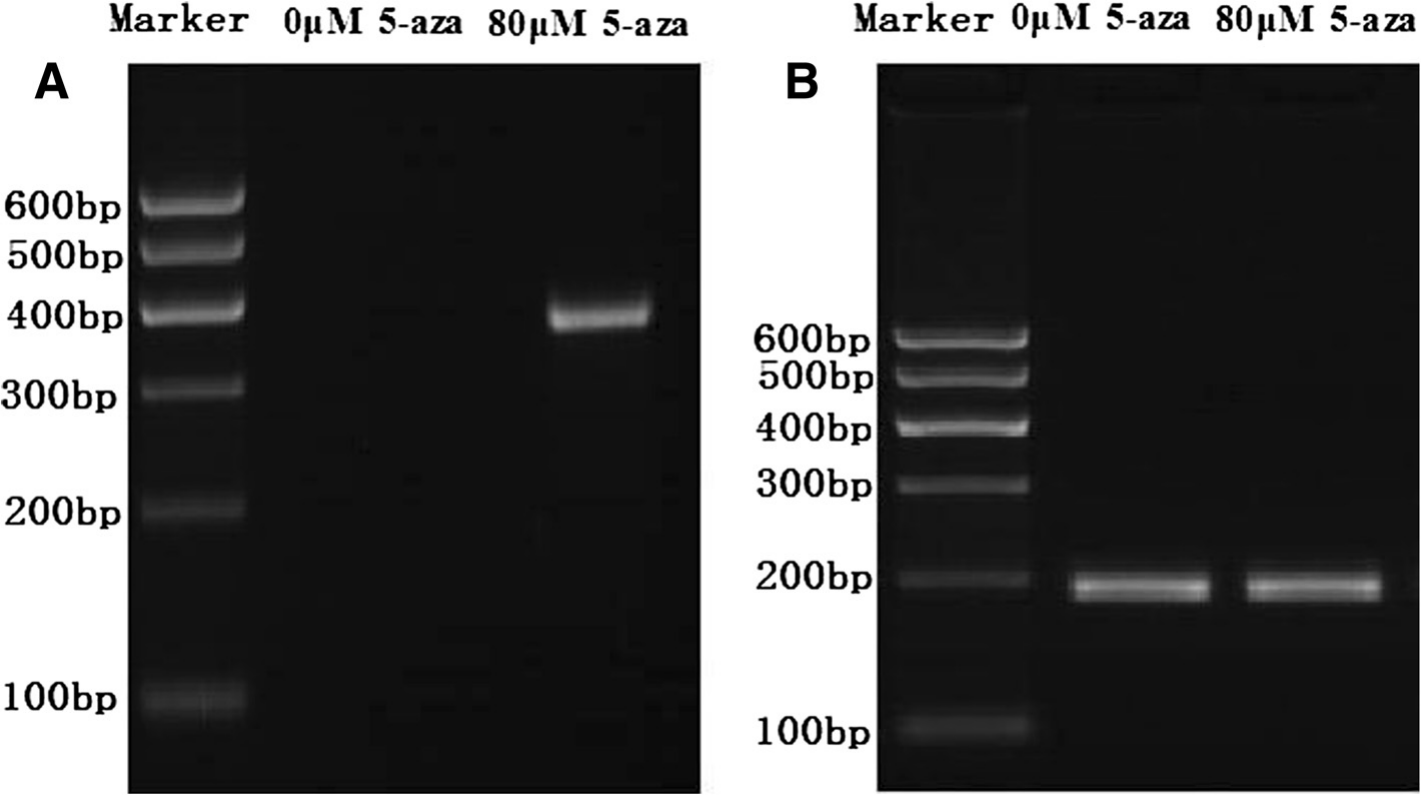
**Effect of 5-aza-cdr on SOX11 mRNA expression in the CNE2 cells. A,** Electrophoretogram of SOX11 mRNA expression; **B,** Electrophoretogram of β-actin mRNA expression. From the electrophoretogram, re-expression of SOX11 mRNA was found in the CNE2 cells after 80 μmol/L 5-aza-cdr treated.

**Figure 8:**
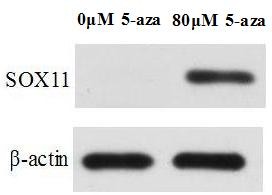
**Effect of 5-aza-cdr on SOX11 protein expression in the CNE2 cells.** The result showed that re-expression of SOX11 protein was found in the CNE2 cells after 80 μmol/L 5-aza-cdr treated.

In order to detect the effect of 5-aza-cdr on expression of SOX11 mRNA and protein in the CNE2 cells, 10^5^ CNE2 cells were seeded onto 6-well plates in each well, and treated the cells using the same methods above. CNE2 cells in 6-well plates were digested with 0.25% trypsin after incubating 48 hours. RT-PCR and Western Blot results showed that re-expression of SOX11 mRNA and protein was found after treating with 5-aza-cdr in CNE2 cells (Figures 7 and 8).

## Discussion

The transcription factor SOX11 plays an important role in embryonic development of the central nervous system and in developing neuron growth and survival as well as recovery of adult neurons following injury tissue [9,10,14]. Several studies have recently demonstrated that SOX11 is up-regulated in various solid tumors, such as lymphoid neoplasms [15], gliomas and epithelial ovarian tumors[11,12]. Brennan [12] revealed that a strong nuclear expression of SOX11 in epithelial ovarian cancer, which correlated with a prolonged recurrence-free survival. So, he suggested that SOX11 plays a functional role in regulation of tumor growth. Hide detected that over-expression of SOX11 prevents tumorigenesis of human glioma initiating cells [16]. Gustavsson found that SOX11 expression can be epigenetically silenced through DNA methylation in a subset of B cell malignancies [17]. In this study, the expression and methylation status of SOX11 gene was detected in nasopharyngeal carcinoma and chronic inflammation tissues of pharynx nasalis. We found that weak expression of SOX11 correlate with methylation of SOX11 gene in nasopharyngeal carcinoma tissues.

Epigenetic mechanism of gene included DNA methylation, histone modifications and RNA interference. DNA methylation is the main epigenetic event in humans, and changes in the DNA methylation pattern play an important role in tumorigenesis [18]. In recent years, the study of tumor suppressor gene promoter methylation has become an important content in occurrence and development of cancer. Many studies have demonstrated that multiple cancer-related genes promoters are frequently methylated in a variety of human cancers [19-21]. DNA methylation is a reversible biochemical modification [22]. The transcriptional inactivation of tumor suppressor gene caused by CpG island methylation can be reversed with DNA methyltransferase inhibitor (5 - aza-2'-deoxycytidine). The reversal (CpG island demethylation) can restore the expression of tumor suppressor gene, and then inhibit cell proliferation and tumor growth [23]. Therefore, restoring the expression of tumor suppressor genes by using DNA methyltransferase inhibitors has become one of the new means of cancer gene therapy. The previous study demonstrated that loss of DAPK expression is associated with aberrant promoter region methylation in nasopharngeal cancer cell line(CNE2) and laryngeal cancer cell line(Hep-2), 5 - aza-2'-deoxycytidine may reactivate DAPK genes silenced by promoter region hypermethylation and can slow the growth of Hep-2 cells and CNE2 cells in vitro and in vivo [24,25]. In the study, the changes of growth and invasion of cells have been detected after being treated with 5 - aza-2'-deoxycytidine in CNE2 cells.The data showed that the inhibition of CNE2 cell growth increased with the increase of drug concentration, invasive capacity of CNE2 cells was significantly decreased, re-expression of mRNA and protein of SOX11 was detected, and SOX11 gene was demethylated after treating with 5-aza-cdr in the CNE2 cells. These results showed that re-expression of SOX11 mRNA and protein may be one of the factors which decrease the growth and invasion capacity of CNE2 cells. Because 5 - aza-2'-deoxycytidine is a DNA methyltransferase inhibitor. It can be reversed the methylated gene in the course of DNA copy.

Nasopharyngeal carcinoma is a common tumor in head and neck. The rradiotherapy is main treatment of nasopharyngeal carcinoma. In recent years, although the technique and equipment of radiotherapy progressive updating, but the therapeutic effect of nasopharyngeal carcinoma is not greatly improved. It is because pathogenesis of nasopharyngeal carcinoma is not very clear. Many studies have explored the pathogenesis and therapeutic effect of nasopharyngeal carcinoma. Most of these studies are genetics and epigenetics. The study of tumor suppressor gene promoter methylation is paid increasing attention. In previous study, we treated CNE2 cells using 5 - aza-2'-deoxycytidine, its proliferation and growth were significantly inhibited, and re-expression of DAPK gene silenced through DNA methylation was found in CNE2 cells [24]. In the present study, after CNE2 cells were treated with 5 - aza-2'-deoxycytidine, SOX11 gene was demethylated and re-expressed, the growth and invasion of CNE2 cells were inhibited. The growth and invasion inhibition of CNE2 cells is probably associated with re-expression of various tumor suppressor genes. The SOX11 gene is one of those tumor suppressor genes. Therefore, SOX11 gene methylation may play a role in growth and invasion of nasopharyngeal carcinoma cells.

## Conclusions

In conclusion, the data provides a comprehensive characterization of the epigenetic mechanisms about SOX11 deregulation in nasopharyngeal carcinoma. No or weak expression of SOX11 gene was detected in some nasopharyngeal carcinoma tissues with DNA methylation. Strong expression of SOX11 gene was found in chronic inflammation tissues of pharynx nasalis and some nasopharyngeal carcinoma tissues with DNA unmethylation. After CNE2 cells were treated with 5 - aza-2'-deoxycytidine, SOX11 gene expression was recovered,and growth and invasion of CNE2 cells were inhibited. It showed that SOX11 expression may be one of the factors that decrease the growth and invasion capacity of CNE2 cells. In a word,additional studies are required to elucidate which is the functional role of the illegitimate SOX11 expression in nasopharyngeal carcinoma.

## Abbreviations

SOX: SRY box-containing; NPC: Nasopharyngeal carcinoma; MSP: Methylation-specific PCR; 5-aza-cdr: 5-aza-2'-deoxycytidine.

## Competing interests

The authors declare that they have no competing interest.

## Authors’ contributions

The work presented here was carried out in collaboration between all authors. SZ and SL defined the research theme, designed methods and experiments; J-LG carried out the laboratory experiments, analyzed the data, interpreted the results and wrote the paper. All authors read and approved the final manuscript.
